# A New Zenith Tropospheric Delay Grid Product for Real-Time PPP Applications over China

**DOI:** 10.3390/s18010065

**Published:** 2017-12-27

**Authors:** Yidong Lou, Jinfang Huang, Weixing Zhang, Hong Liang, Fu Zheng, Jingnan Liu

**Affiliations:** 1 GNSS Research Center, Wuhan University, Wuhan 430079, China; ydlou@whu.edu.cn (Y.L.); jfhuang@whu.edu.cn (J.H.); fuzhgnss@whu.edu.cn (F.Z.); jnliu@whu.edu.cn (J.L.); 2 Collaborative Innovation Center of Geospatial Technology, Wuhan University, Wuhan 430079, China; 3 Meteorological Observation Centre of China Meteorological Administration, Beijing 100081, China; liangh@camscma.cn

**Keywords:** BDS/GPS, zenith tropospheric delay, real-time precise point positioning, ECMWF, CMONOC

## Abstract

Tropospheric delay is one of the major factors affecting the accuracy of electromagnetic distance measurements. To provide wide-area real-time high precision zenith tropospheric delay (ZTD), the temporal and spatial variations of ZTD with altitude were analyzed on the bases of the latest meteorological reanalysis product (ERA-Interim) provided by the European Center for Medium-Range Weather Forecasts (ECMWF). An inverse scale height model at given locations taking latitude, longitude and day of year as inputs was then developed and used to convert real-time ZTD at GPS stations in Crustal Movement Observation Network of China (CMONOC) from station height to mean sea level (MSL). The real-time ZTD grid product (RtZTD) over China was then generated with a time interval of 5 min. Compared with ZTD estimated in post-processing mode, the bias and error RMS of ZTD at test GPS stations derived from RtZTD are 0.39 and 1.56 cm, which is significantly more accurate than commonly used empirical models. In addition, simulated real-time kinematic Precise Point Positioning (PPP) tests show that using RtZTD could accelerate the BDS-PPP convergence time by up to 32% and 65% in the horizontal and vertical components (set coordinate error thresholds to 0.4 m), respectively. For GPS-PPP, the convergence time using RtZTD can be accelerated by up to 29% in the vertical component (0.2 m).

## 1. Introduction

In GNSS data processing, in order to avoid the rank defect issue, slant tropospheric delays along the signal paths between visible satellites and the receiver are usually mapped into the zenith direction (i.e., zenith total delay or ZTD) with a mapping function. ZTD can be divided into zenith hydrostatic delay (ZHD) and zenith non-hydrostatic delay which is always named as zenith wet delay (ZWD). ZHD, which accounts for about 90% of the ZTD, can be precisely calculated on the bases of models given the observed meteorological parameters. ZWD or the correction to *a priori* ZWD which will assimilate ZTD residual errors, however, usually needs to be estimated in the data processing because of the complex variations of water vapor in both time and space. There are some empirical tropospheric delay estimation models, among which Hopfield model [1] and Saastamoinen model [2] are most commonly used. These two models can provide *a priori* ZTD with accuracies of decimeters to sub-decimeters when precise meteorological parameters are available.

Great efforts have also been made to develop new *a priori* models with good performances even without precise meteorological measurements, such as the UNB models which were initially developed for users of the Wide Area Augmentation System (WAAS). In North America, the bias of ZTD derived from UNB3 is 2.0 cm and decreases to −0.5 cm for UNB3m [3,4]. The deviations of ZTD derived from UNB3m, however, can reach 6.1 cm in high altitude regions [5]. The EGNOS model, which is a simplified version of UNB3, can provide ZTD with equivalent accuracy compared to the meteorological-parameter-based Hopfield or Saastamoinen model [6]. Li et al. developed a new global tropospheric zenith delay model (IGGtrop) based on the NCEP reanalysis data. According to their assessment using 125 International GNSS Service (IGS) stations, the mean bias and Root Mean Square (RMS) of ZTD are about 0.8 and 4.0 cm, respectively [7,8]. Yao et al. analyzed the temporal and spatial variations of ZTD based on Global Geodetic Observing System (GGOS) atmosphere data and built the GZTD model using spherical harmonic function. Compared to IGGtrop, GZTD has equivalent accuracies but less model parameters [9].

A wide range of mapping functions has also been developed in the past. There are mainly two kinds of mapping functions. The first one is the so-called empirical mapping function which only needs the epoch time and stations approximate coordinates as inputs, such as the Niell Mapping Function (NMF) [10], the Global Mapping Function (GMF) [11] and mapping functions derived from Global Pressure and Temperature 2 (GPT2/GPT2w) [12,13]. These mapping functions were developed based on historical radiosondes or reanalysis products, which do not need external data in geodetic data processing. The second kind of mapping functions, such as Isobaric Mapping Functions (IMF) [14] and Vienna Mapping Functions (VMF1) [15], are derived from the operational analysis fields of numerical weather models at the epoch of the observations. They are theoretically more accurate than empirical mapping functions, but they rely on external data source.

Due to the strong correlation between ZTD and the vertical position, Precise Point Positioning (PPP) processing usually needs several tens of minutes to separate these two parameters for convergence, which significantly limits its real-time applications. The convergence time is much longer for users only using Chinese BeiDou Navigation Satellite System (BDS) due to the current satellite constellation, namely most of the satellites are geostationary satellites (GEO) and inclined geostationary satellites (IGSO) with slow changes of geometry relative to ground users. If the accurate *a priori* ZTD are available, ZTD can be fixed or strongly constrained in PPP processing and the convergence can be efficiently accelerated [16,17]. However, the current *a priori* ZTD models mentioned above can only provide ZTD with accuracy not better than 4 cm, which is not accurate enough for the real-time PPP [18]. The Numerical Weather Prediction models (NWP) can also be used for retrieving real-time ZTD. For example, Andrei et al. evaluated the accuracy of hourly ZTD derived from the Global Data Assimilation System (GDAS) numerical weather model (NWM) [19]. The average RMS of the GDAS NWM-derived ZTD is about 3 cm by comparing with IGS PPP ZTD at 18 IGS stations over 1.5 years. Lu et al. made comparisons of ZTD between ECMWF analysis and IGS solutions over one month and found an average RMS of about 1.5 to 2.2 cm which depends on the latitudes [20,21]. However, the NWP products are usually in the time resolution of several hours (e.g., typically from 1 to 6 h), which is not sufficient enough for high-rate real-time applications, especially, under some fast-changing weather conditions.

With the development of PPP-RTK technique, the precise ZTD derived from regional Continuously Operating Reference Stations (CORS) network have been used to interpolate ZTD for users, providing more accurate real-time ZTD than *a priori* empirical models. Li et al. proposed the modified linear combination model (MLCM) for zero-difference tropospheric interpolation [22]. Zhang et al. improved MLCM and addressed the spatial regression model with altitude difference constraint, with interpolated ZTD RMS of about 6~7 mm in flat areas and about 18 mm in undulate areas [23]. However, these models are only valid for users in regions with CORS network available, which is usually at the scale of a city or a province.

In recent two years, there are also some publications focusing on wide-area real-time tropospheric product developments, such as [24]. They utilized real-time GNSS stations and IGGtrop ZTD models to generate grid-based ZTD/ZWD products. In this study, we will present a new inverse scale height model and apply it to real-time ZTD solutions at GPS stations at a national scale of China to generate the real-time ZTD grid product for real-time PPP users. The data sources and processing strategy will be described in Section 2, and the construction of the real-time ZTD grid product will be then depicted in Section 3. The real-time ZTD grid product will be evaluated by comparisons with post-processing ZTD and by PPP convergence tests in Section 4. Finally, some discussions and conclusions will be given in Section 5.

## 2. Data and Processing Strategy

In this section, the reanalysis product (ERA-Interim) and the ZTD estimation method as well as the used GPS data and data processing strategy will be described.

### 2.1. Reanalysis Data and ZTD Estimation Method

ERA-Interim is the reanalysis product from the European Centre for Medium-Range Weather Forecasts (ECMWF). It provides 6-hourly data with the horizontal resolution of 0.75° × 0.75°, and 37 vertical pressure levels from 1000 hPa up to 0.1 hPa. Fields including geopotential, temperature and specific humidity on each level were used.

Similar to the method used in [19,25], the final ZTD at a given location is the sum of two sections, namely the ZTD section from the location to the top level (estimated by integration method) (*ZTD_level_*) and the hydrostatic section above the top level (there is almost no water vapor above this level) of reanalysis data (estimated by Saastamoinen model) (*ZHD_top_*). The value of *ZHD_top_* depends on the pressure on the top level of reanalysis product. For reanalysis products like NCEP/NCAR, the top level is 1 hPa, which corresponds to about 2 mm of *ZHD_top_* according to Saastamoinen model, while for ERA-Interim used in this work, the top level pressure is only 0.1 hPa, corresponding to about 0.2 mm of *ZHD_top_*, which is far below the GPS ZTD estimation errors. Although ignoring *ZHD_top_* will not introduce significant biases to ERA-Interim-based ZTD when comparing with GPS, in order to estimate ZTD as accurate as possible, we still write the final ZTD as:

(1) $$ZTD=ZHD_{top}+ZTD_{level}$$

(2) $$ZHD_{top}=0.0022768\cdot \frac{P_{top}}{1-0.00266\cdot \cos(2\varphi )-0.00028\cdot h_{top}}$$

(3) $$ZTD_{level}=10^{-6}\int_{h_{given}}^{h_{top}}Ndh=10^{-6}\sum_{i}N_{i}\Delta s_{i}$$

where *P_top_* is the atmospheric pressure at the top level in hPa, φ is the latitude, *h_top_* is the altitude of the top level in meters, *h_given_* is the given location altitude, and *N* is the refraction which can be calculated by:

(4) $$N=\frac{k_{1}(P-e)}{T}+(\frac{k_{2}e}{T}+\frac{k_{3}e}{T^{2}})$$

(5) e=qP/0.622

where *k*_1_ = 77.604 K/hPa, *k*_2_ = 64.79 K/hPa, *k*_3_ = 377,600.0 K^2^/hPa [26]. The *P* and *e* denote the atmospheric pressure and water vapor partial pressure in hPa, respectively. *T* denotes the air temperature in K, and *q* is the specific humidity in kg/kg.

For GPS antenna level above the lowermost level of the reanalysis product, the temperature at the antenna level was estimated by linear interpolation, while for GPS antenna level below the lowermost level, the temperature at the antenna level was estimated by linear extrapolation, assuming a constant temperature lapse rate of −6.5 K/km^−1^, and constant relative humidity of the value at the lowermost level. The hydrostatic and ideal gas equations were used to adjust pressure from reanalysis to the GPS antenna level as described in [27].

In order to evaluate the accuracy of ZTD derived from ERA-Interim, IGS final ZTD products at six IGS stations located in China were taken as references for comparisons for one year (2014). The mean bias as shown in Table 1 is 0.36 cm and the RMS is 1.40 cm, which is basically consistent with the results in [28].

### 2.2. GPS Data and the Processing Strategy

The observation equations of the ionospheric-free code and phase combinations in GPS data processing can be expressed as:

(6) $$\left\{ \begin{array}{l} P_{r}^{s}(t)=|{\vec{X}}_{r}(t)-{\vec{X}}^{s}(t)|+\Delta t_{r}(t)-\Delta t^{s}(t)+\Delta trop_{r}^{s}(t)+\varepsilon_{r}^{s}(t)\\ L_{r}^{s}(t)=|{\vec{X}}_{r}(f)-{\vec{X}}^{s}(f)|+\Delta t_{r}(t)-\Delta t^{s}(t)+\Delta trop_{r}^{s}(t)+\lambda \cdot N_{r}^{s}+e_{r}^{s}(t) \end{array}\right.$$

where $P_{r}^{s}(t)$ and $L_{r}^{s}(t)$ denote the code and carrier phase observations between satellite *s* and receiver *r* at time *t*. ${\vec{X}}_{r}$ represents the receiver position vector with Phase Center Correction (PCO), Phase Center Variation (PCV) and tide loading effects considered, while ${\vec{X}}^{s}$ represents satellite position vector with PCO and PCV corrected. $\Delta t_{r}$ and $\Delta t^{s}$ denote receiver and satellite clock errors, respectively. ε and e are the residual noises and un-modeled errors. Δtrop is the total tropospheric delay along the signal path which can be written as:

(7) $$\Delta trop=mf_{h}\cdot ZHD+mf_{nh}\cdot ZWD+mf_{G}\cdot (G_{ns}\cdot \cos(a)+G_{ew}\cdot \sin(a))$$

where *mf_h_* and *mf_nh_* denote the hydrostatic and non-hydrostatic mapping functions and *mf_G_* represents the gradient mapping function. *G_ns_* and *G_ew_* represent the two horizontal gradients in north-south and east-west component, respectively, and *a* is the azimuth of the line of sight.

The Crustal Movement Observation Network of China (CMONOC) is the national scientific infrastructure aiming to monitor the current intra-plate deformation in China, using space geodetic techniques such VLBI, SLR and GPS [29]. Currently, it comprises about 260 continuous GPS stations with the average station separation of about 200 kilometers. GPS data from CMONOC covering the period of four months (Jan., Apr., Jul. and Oct.) in four seasons in 2015 with time interval of 30 s were processed using Position And Navigation Data Analyst (PANDA) [30,31,32,33] in simulated real-time PPP mode. Simulated real-time mode means that the data are processed only in forward direction but read from Receiver Independent Exchange Format (RINEX) files instead of the real-time stream, and all time delays existing in real-time situations are neglected. The archived real time GPS orbit and clock products from IGS (ftp://cddis.gsfc.nasa.gov/gnss/products/rtpp/) (i.e., IGS01) were used [34]. ZHD and ZWD were first calculated by *a priori* Saastamoinen model and the corrections to ZWD, which can also assimilate ZHD residual errors, were estimated as random walk parameters. The final ZTD were acquired by summing over ZWD corrections and *a priori* ZHD and ZWD. The global pressure and temperature 2 wet (GPT2w) model [13] was used in the processing. Tropospheric gradients in the north-south and east-west directions were estimated at 12 h intervals. The details of the processing strategy are summarized in Table 2.

Taking ZTD estimated in post-processing mode (i.e., IGS final orbits and clocks were used and parameters were estimated by least squares method) as references, the bias and RMS of real-time ZTD errors at all stations in CMONOC for four months in different seasons are summarized in Table 3 where the mean bias and RMS are about 0.25 cm and 1.18 cm, respectively.

## 3. Real-Time ZTD Grid Product Generation

In this section, the vertical variations of ZTD derived from ERA-Interim will be first analyzed and the inverse scale height model is determined and then applied to real-time ZTD solutions at GPS stations at a national scale over China for the generation of the real-time ZTD grid product.

### 3.1. Inverse Scale Height Model

**Figure 1:**
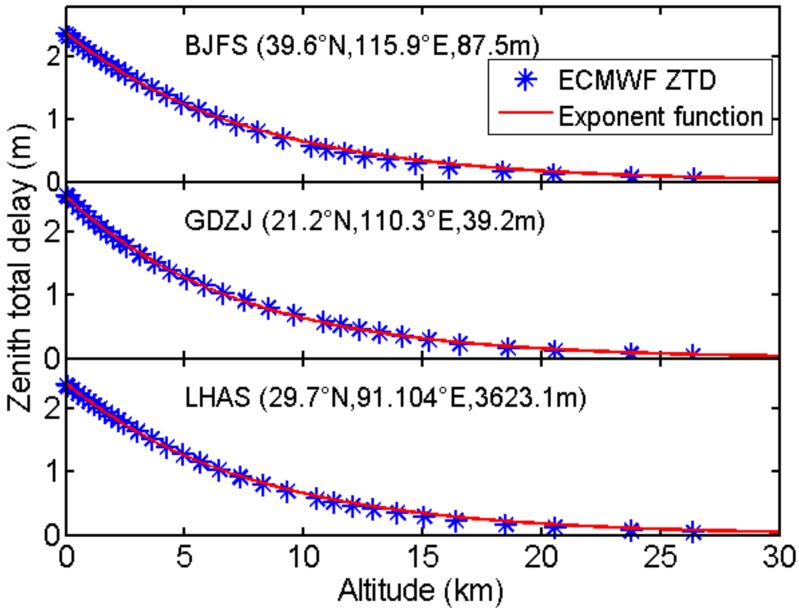
Profiles of ZTD at the nearest grid points of the GPS stations, BJFS, GDZJ and LHAS at UTC 0, 1 April 2014.

Figure 1 presents the vertical profiles of ZTD derived from ERA-Interim at the nearest grid points from station BJFS, GDZJ and LHAS. These three stations locate in different areas in China with significantly different latitudes and altitudes. The ZTD profiles show exponentially or near exponentially decreasing trends with altitudes.

Similar to [8], the exponential function for ZTD vertical variations can be expressed as:

(8) $$ZTD(h)=ZTD_{0}\cdot \exp(-\frac{1}{H}\cdot h)$$

where *ZTD*_0_ is zenith tropospheric delay at MSL, *H* is the scale height, and *h* denotes the location altitude above MSL.

Equation (8) can also be modified to convert ZTD at a given height (*h*_1_) to the required height (*h*) as:

(9) $$ZTD(h)=ZTD_{1}\cdot \exp(-\frac{1}{H}\cdot (h-h_{1}))$$

where *ZTD*_1_ is zenith tropospheric delay at the given height *h*_1_.

Considering the practical situations, we rarely do PPP for GPS receivers which are located vertically too far away from the ground surface. Therefore, we designed two ZTD fitting tests here. In the first test, the whole ZTD profiles (from 1000 hPa to 0.1 hPa) at each grid point were fitted using (9), while in the second test only ZTD at altitudes within 3 km from the terrain surface were used. The bias and standard deviation (STD) as well as the maximum and minimum values of fitting residuals are summarized in Table 4. The comparisons show that in the terrain ±3 km test the fitting residuals are much smaller with STD of 0.42 cm. Therefore, in the following sections, we will only fit ZTD within 3 km from the terrain surface (surface altitude are acquired from ERA-Interim products) to build the inverse scale height (1/*H*) model.

### 3.2. Inverse Scale Height Modeling

Before modeling the inverse scale height (1/*H*), we did a sensitivity test first to get a thumb rule regarding the relationship between 1/*H* and ZTD errors. ERA-Interim products for one day (1 January 2014) are used to estimate 1/*H* at each grid point in China. The average value of 1/*H* at all grid points is 1.35 × 10^−^^4^ m^−^^1^, with minimum and maximum values of 1.21 × 10^−^^4^ m^−^^1^ and 1.49 × 10^−^^4^ m^−^^1^, respectively. The average value of 1/*H* instead of the actual 1/*H* was then used in (8) to estimate ZTD at each grid point and the differences between the estimated ZTD and the actual ZTD were calculated. According to the statistics, we found a change of 1/*H* with 0.1 × 10^−^^4^ m^−^^1^ corresponds to about 2.8 cm in ZTD.

**Figure 2:**
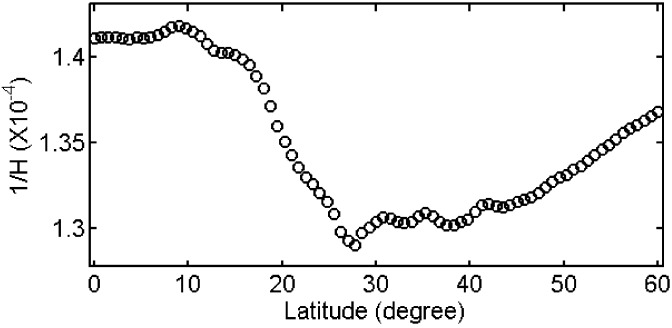
Distribution of inverse scale height with latitudes.

Figure 2 presents the distribution of mean 1/*H* at different latitudes over China. The amplitude is larger than 0.1 × 10^−4^ m^−^^1^, which corresponds to about 2.8 cm ZTD according to the sensitivity test. In addition, according to our statistics, the differences of 1/*H* at different longitudes in the same latitude can reach 0.2 × 10^−4^ m^−^^1^ (corresponds to 5.6 cm of ZTD). Obviously, 1/*H* has considerable spatial variations, which needs to be taken into account when modeling 1/*H*.

**Figure 3:**
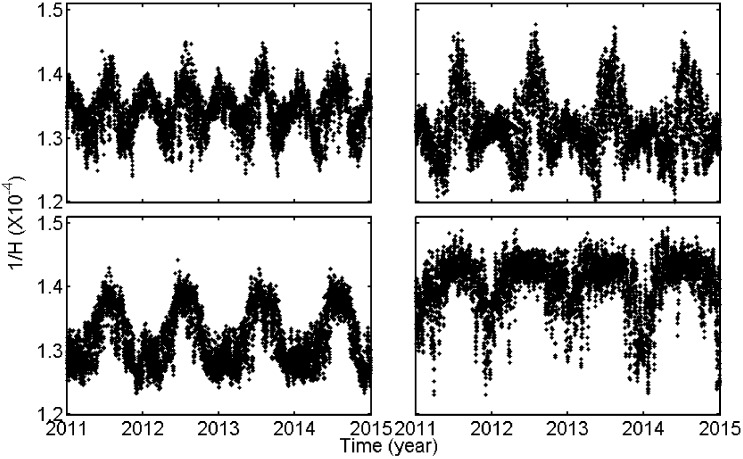
The 4-year (2011~2014) time series of parameter 1/*H* at the nearest grid points of four GPS stations.

**Figure 4:**
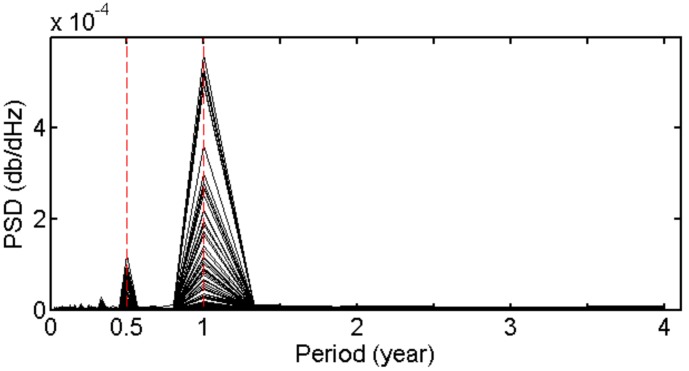
Power Spectral Density (PSD) of 1/*H* time series at all grid points over China.

The temporal variations of 1/*H* were studied on the bases of 4-year (2011~2014) time series of 1/*H*. As an example, the time series of 1/*H* at nearest grid points from four GPS stations (HLHG, BJFS, LHAS and GDZJ) are shown in Figure 3 where we can find obvious periodical signals. The Power Spectral Density (PSD) of 1/*H* time series at all grid points over China are presented in Figure 4, where we can find the time series mainly contain annual and semi-annual periodical signals. Therefore, we used the model as expressed in (10) to fit 1/*H* at each grid point:

(10) $$\begin{array}{ll} 1/H= & a_{0}+a_{1}\cos(2\pi \frac{doy}{365.25})+a_{2}\sin(2\pi \frac{doy}{365.25})+\\ & a_{3}\cos(4\pi \frac{doy}{365.25})+a_{4}\sin(4\pi \frac{doy}{365.25}) \end{array}$$

where *a*_0~4_ are coefficients.

Based on the above analysis, ZTD time series covering the period from 2011 to 2014 at each grid point (0.75° × 0.75° resolution) derived from ERA-Interim were used to estimate 1/*H* by (8). The inverse scale height time series at each grid point were then fitted by (10) to generate a coefficient (*a*_0_, *a*_1_, *a*_2_, *a*_3_, *a*_4_) table. The Mean Absolute Error (MAE) and RMS of fitting residuals are 0.03 × 10^−4^ m^−^^1^ and 0.039 × 10^−4^ m^−^^1^, corresponds to 0.84 cm and 1.09 cm of ZTD, respectively.

### 3.3. Real-Time ZTD Grid Product Generation Procedure

ZTD at GPS stations in CMONOC were estimated in the simulated real-time mode using PANDA package with 5 min interval. For each grid point with horizontal resolution of 0.75° × 0.75° covering the area of 10° N~60° N and 70° E~140° E, the GPS stations within 1000 km near the grid point were selected. ZTD at these GPS stations were converted from the station altitude to the grid point surface altitude using the above inverse scale height model which can be expressed as:

(11) $$ZTD_{c_{i}}(h_{g})=\frac{ZTD_{c_{i}}(h_{c_{i}})}{\exp(-{\left(1/H\right)}_{c_{i}}\cdot h_{c_{i}})}\cdot \exp(-{\left(1/H\right)}_{c_{i}}\cdot h_{g})=ZTD_{c_{i}}(0)\cdot \exp(-{\left(1/H\right)}_{c_{i}}\cdot h_{g})$$

where the subscripts of *c_i_* and *g* denote the station and the grid point, respectively.

The inverse distance weighted (IDW) method was then applied to calculate the ZTD at the grid point as,

(12) $$ZTD_{g}(h_{g})=p_{1}\cdot ZTD_{c_{1}}(h_{g})+p_{2}\cdot ZTD_{c_{2}}(h_{g})+\ldots +p_{n}\cdot ZTD_{c_{n}}(h_{g})$$

where *p_i_* is the weight coefficient for each station. In order to make the product easy to use, the ZTD at the grid point surface were eventually converted to MSL using the inverse scale height model.

For users, ZTD at the nearest four grids around the user were converted from MSL to the user’s altitude by inverse scale height model and then IDW method were used for horizontal interpolation to calculate the ZTD at user’s location.

## 4. Real-Time ZTD Grid Product Evaluations

In this section, the accuracy of the generated real-time ZTD grid product (RtZTD) will be first assessed at test stations by comparing with post-processing ZTD solutions. Simulated real-time PPP convergence tests will also be carried out to evaluate the improvements for PPP users by using RtZTD.

### 4.1. Accuracy Assessments

**Figure 5:**
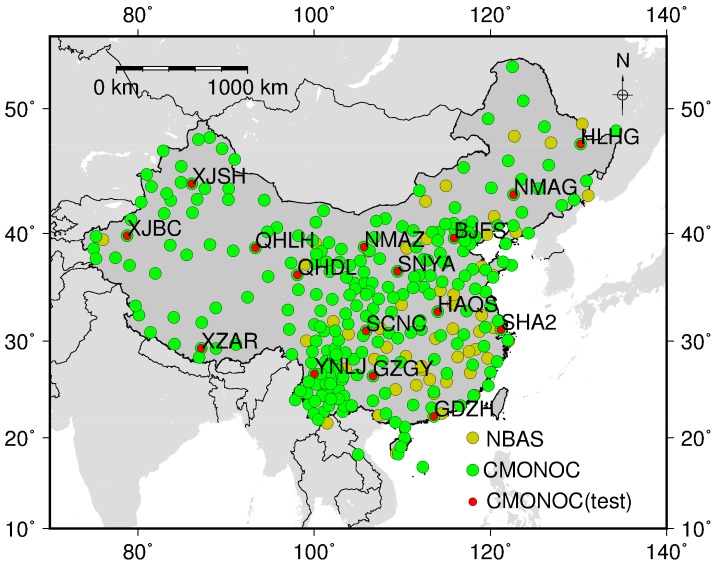
The distribution of the GPS stations: Yellow circles denote GPS stations from NBASS; Green circles denote CMONOC base stations and red circles denote CMONOC test stations.

GPS Data at 260 CMONOC GPS stations (Figure 5) in four months (Jan., Apr., Jul. and Oct.) during year 2015 were processed, among which 16 stations were selected as test stations, and the other stations were taken as base stations for real-time ZTD grid product generation.

**Figure 6:**
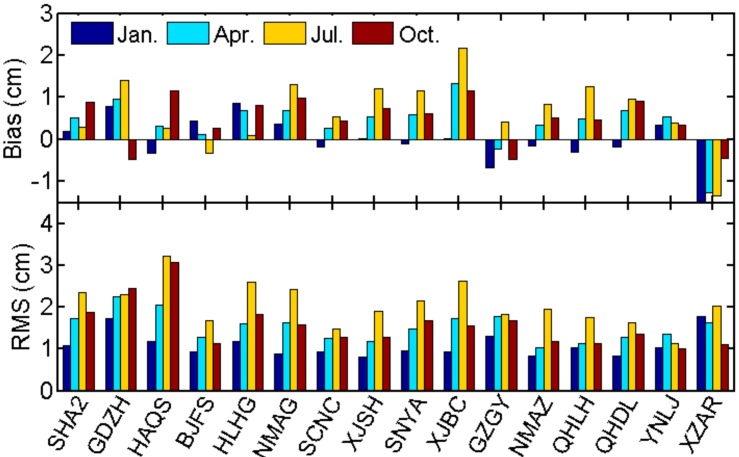
Bias and error RMS of ZTD estimation using RtZTD at test stations in each month (stations are sorted in ascending altitude).

Taking post-processing ZTD as references, the bias and error RMS of ZTD calculated from real-time grid product at 16 test stations are summarized in Table 5. We can find smaller RMS of about 1.09 cm in winter (Jan.) with insignificant bias, but larger RMS of 2.07 cm in summer (Jul.) with bias of 0.66 cm. The bias and error RMS at each test station are presented in Figure 6. The largest bias (~2.2 cm) is found at station XJBC in summer, which may be due to the fact that station XJBC locates at the edge of the CMONOC network with sparse stations around. RMS in summer are usually larger than RMS in other seasons mainly due to larger variations of water vapor content in summer. Overall, according to Table 5 and Figure 6, the generated real-time ZTD grid product based on CMONOC can provide real-time ZTD with bias of 0.39 cm and RMS of 1.56 cm, and the accuracy is not significantly affected by the station altitude.

**Figure 7:**
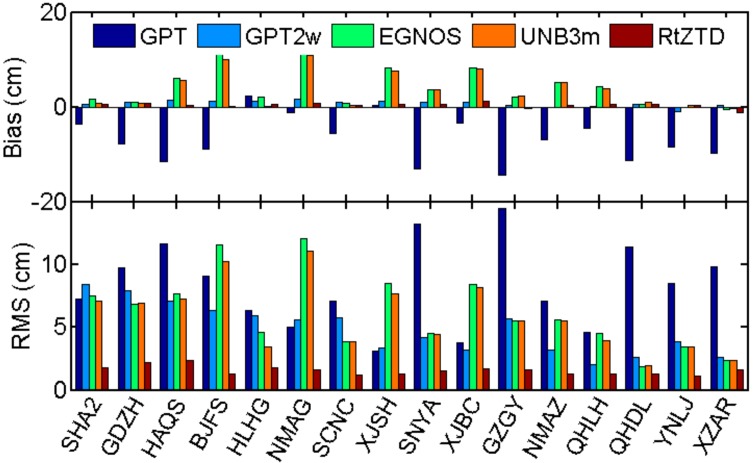
Comparison of GPT, GPT2, EGNOS, UNB3m model and RTZTD (stations sorted in ascending altitude).

Figure 7 presents the comparisons of performances for RtZTD and four widely used empirical ZTD models, including Global Pressure and Temperature model (GPT), GPT2w, UNB3m and EGNOS. ZTD derived from GPT means that the GPT and standard atmosphere parameters were used as inputs in Saastamoinen model. For GPT2w, ZTD is the sum of GPT2w-derived ZWD and ZHD estimated from Saastamoinen model based on GPT2w-derived pressures. From Figure 7 we find that GPT2w is evidently better than GPT, especially for stations with high elevation. The mean bias of UNB3m is about 3.74 cm, which is larger than values in both [5] (0.5 cm) and [8] (1.46 cm). The reason might be that only 16 stations in China were used in Figure 7 while [5,8] used global distributed stations for the assessment. Compared to the empirical models, the improvement by using RtZTD is significant, with bias of 0.39 cm and RMS of only 1.56 cm.

### 4.2. PPP Convergence Tests

RtZTD can provide users with high-accuracy *a priori* ZTD, which can be used to accelerate the PPP convergence. 95 stations with both GPS and BDS observations available from National BDS Augmentation Service System (NBASS) are selected as test stations in PPP convergence tests. All CMONOC stations were used as base stations to generate RtZTD product with a resolution of 0.75° × 0.75° in the simulated real-time mode. The tests cover one week from 4 Apr to 10 Apr 2016 and the GPS/BDS data were processed with 30 s interval. The convergence time of PPP using RtZTD product (denoted as RtZTD) and the traditional PPP (Saastamoinen + GPT2w as *a priori*, and the remaining part is estimated) (denoted as non-RtZTD) will be compared.

**Figure 8:**
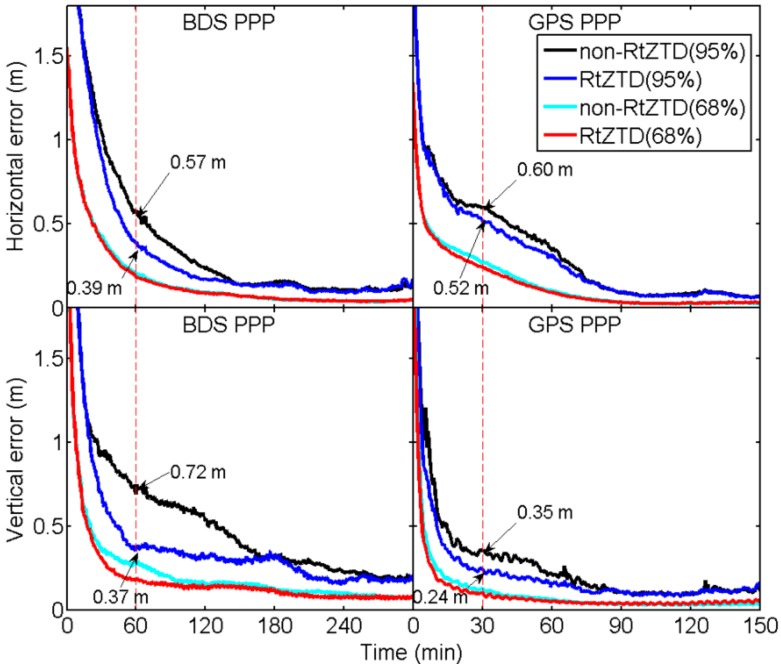
Convergence time comparison for BDS/GPS PPP in horizontal component and vertical component.

For each test station, we calculated the differences between coordinates derived from PPP and the ground-truth (estimated in post-processing mode) in horizontal and vertical components, respectively. Afterwards, at each epoch, the coordinate absolute differences were sorted from lowest to highest. The maximum value among 95% (2σ) or 68% (1σ) of the sorted coordinate differences at each epoch is selected [35] and plotted in Figure 8. As shown in Figure 8, in both 95% and 68% situations, using RtZTD can significantly accelerate the convergence. After 60 and 30 min for BDS- and GPS-PPP, for example, coordinate errors decrease from 0.57 to 0.39 m in horizontal component and from 0.72 to 0.37 m in vertical component for BDS-PPP using RtZTD in 95% situation. For GPS-PPP, the errors decrease from 0.60 to 0.52 m and from 0.35 to 0.24 m in horizontal and vertical components, respectively.

According to the statistics of coordinate error RMS after convergence (the first-three-hour results removed) as shown in Table 6, we set the thresholds for convergence as 0.4 and 0.2 m of coordinate errors for BDS- and GPS-PPP, respectively, in 95% situation, while the thresholds as 0.2 and 0.1 m for BDS and GPS, respectively, in 68% situation.

Table 7 summarizes the PPP convergence time of non-RtZTD and RtZTD cases. For BDS-PPP, the convergence time improved about 32% and 65% in the horizontal and vertical components in 95% situation, respectively, and improved up to 6% and 65% in 68% situation. For GPS-PPP, although there are no significant improvements in the horizontal component, the improvements in the vertical direction are approximately 29% and 25% in 95% and 68% situation, respectively.

## 5. Discussion and Conclusions

The temporal and spatial variations of ZTD with altitude over China were analyzed on the bases of the latest meteorological reanalysis products (ERA-Interim). Results show that the variations of ZTD with altitude are near exponential, and the temporal variations of exponential function coefficients at specific location mainly contain annual and semi-annual periodical signals. An inverse scale height model was then determined and applied to ZTD solutions derived from CMONOC GPS stations in simulated real-time mode to generate the ZTD grid products. Using 16 stations in CMONOC as test stations, the ZTD derived from the ZTD grid product were compared to ZTD estimated in post-processing mode and the average bias and RMS of the ZTD differences are 0.39 cm and 1.56 cm, respectively.

All GPS stations in CMONOC were used to generate RtZTD products in simulated real-time mode, and 95 stations with GPS and BDS observations in NBASS were taken as test stations for PPP convergence tests. Data covering one week were processed and the convergence time of PPP with and without RtZTD products used were compared. Regarding the convergence time, the thresholds for convergence were determined based on coordinate error RMS statistics, i.e., 0.4 and 0.2 m of coordinate errors for BDS- and GPS-PPP, respectively, in 95% statistics, while 0.2 and 0.1 m for BDS and GPS, respectively, in 68% statistics. The results show considerable improvements in convergence time by using RtZTD of about 32% (6%) and 65% (65%) in the horizontal and vertical components for BDS-PPP, and 29% (25%) in vertical direction for GPS PPP in 95% (68%) statistics.

The time delays, including transmission time from reference stations to the data center, data processing time and transmission time from the data center to users, are generally smaller than 10 s in current augmentation systems. For example, the total time delays in the Global Differential GPS (GDGPS) System developed by JPL are smaller than 5 s. Considering the relatively small variations of orbit and clock corrections (<1 cm) and ZTD (<1 mm) in small intervals like 10 s (variations not shown here), these time delays can be safely ignored in real-time applications. This is also the reason why in some satellite-based augmentation system services, e.g., the QZSS L6 signal, which provides centimeter level augmentation services, can broadcast orbit and clock corrections in 30 and 5 s intervals without using any prediction models, and why IGS centers can estimate ZTD as constants in 5 min intervals. Therefore, although all real-time tests in this work were carried out in the simulated mode with time delays ignored, the conclusions can provide important references to real-time applications. As more and more real-time GPS stations are available in China, we can expect better and more robust results in the near future.

## Figures and Tables

**Table 1:** The bias and RMS of differences between IGS final ZTD products and ZTD derived from reanalysis for year 2014 (unit: cm).

Station Name	Bias	RMS
BJFS	0.29	1.25
CHAN	−0.19	1.09
LHAZ	0.76	1.43
SHAO	−0.53	0.94
URUM	1.94	2.30
WUHN	−0.14	1.38
Mean	0.36	1.40

**Table 2:** GPS data processing strategy.

Measurement Model	
Basic observables	Ionosphere-free combinations of code and phase measurements on L1/L2 for GPS
Sampling rate	30 s
Elevation cutoff angle	7°
Weighting	A priori precision of 0.03 cycles and 3.0 m for raw phase and code, respectively.
	Elevation-dependent, 1 for E > 30°, otherwise 2sin(E)
Phase center corrections	igs08.atx
Phase wind up	Corrected
Tropospheric a-priori model	GPT2w
Station displacement corrections	IERS 2003 conventions
DCBs	Corrected with products provided by CODE
Relativistic effects	Corrected
**Stochastic Model**	
Estimation method	Real-time mode: Square Root Information Filter (SRIF)
	Post-processing mode: Least Square Adjustments
Station coordinates	Fixed
Satellite orbit and clocks	Real-time mode: IGS archived real-time products (IGS01)
	Post-processing mode: IGS final post-processing products
Receiver clocks	White noise with a unit weight variance of 9000 m
ZWD corrections	Real-time mode: Random walk process with a constraint of 20 mm/√h
	Post-processing mode: Piece-wise constants with 5 min interval with a constraint of 20 mm/√h
Tropospheric gradients	Piece-wise constants with 12 h interval with a constraint of 5 mm/√h
Ambiguity	Float

**Table 3:** Bias and RMS of real-time ZTD errors (unit: cm).

	Jan.	Apr.	Jul.	Oct.	Mean
Bias	0.05	0.26	0.28	0.41	0.25
RMS	1.07	1.22	1.21	1.23	1.18

**Table 4:** Statistics of ZTD fitting residuals on 1 January 2014 (unit: cm).

Fitting Layer	Bias	STD	Max	Min
All	−0.39	1.69	2.48	−3.26
Terrain ±3 km	−0.001	0.42	0.59	−0.55

**Table 5:** Mean bias and error RMS of ZTD estimation using RtZTD at 16 test stations in different months (unit: cm).

	Jan.	Apr.	Jul.	Oct.	Mean
Bias	−0.03	0.41	0.66	0.49	0.39
RMS	1.09	1.52	2.07	1.57	1.56

**Table 6:** RMS of the positioning error after convergence (the first-three-hour results removed) (unit: cm).

	Non-RtZTD PPP				RtZTD PPP			
	H (95%)	H (68%)	V (95%)	V (68%)	H (95%)	H (68%)	V (95%)	V (68%)
BDS	13.91	4.36	22.26	8.78	13.60	4.53	19.28	7.70
GPS	9.41	2.30	10.81	3.47	9.34	2.47	11.92	4.86

**Table 7:** Convergence time comparisons of non-RtZTD and RtZTD PPP (unit: min) *.

	Non-RtZTD PPP				RtZTD PPP			
	H (95%)	H (68%)	V (95%)	V (68%)	H (95%)	H (68%)	V (95%)	V (68%)
BDS	84.5	62	149.5	87.5	57.5	58	52	30.5
GPS	73.5	60.5	62.5	36	71.5	57.5	44.5	27

* Note: Different thresholds are used for BDS- and GPS-PPP.

## References

[1] Hopfield H.S. (1971). Tropospheric effect on electromagnetically measured range: Prediction from surface weather data. Radio Sci..

[2] Saastamoinen J. (1972). Atmospheric correction for the troposphere and stratosphere in radio ranging of satellites. Use Artifi. Satell. Geod..

[3] Collins J.P., Langley R.B., LaMance J. Limiting factors in Tropospheric Propagation Delay Error Modelling for GPS Airborne Navigation. Proceedings of the Annual Meeting of the Institute of Navigation.

[4] Collins J.P., Langley R.B. (1997). A Tropospheric Delay Model for the User of the Wide Area Augmentation System.

[5] Leandro R., Santos M., Langley R. UNB neutral atmosphere models: Development and performance. Proceedings of the 2006 National Technical Meeting of The Institute of Navigation, Institute of Navigation.

[6] Nigel P., Alan D., Wu C. (2001). Assessment of EGNOS Tropospheric Correction Model. J. Navig..

[7] Li W., Yuan Y., Qu J., Li H., Li Z. (2012). A new global zenith tropospheric delay model IGGtrop for GNSS applications. Chin. Sci. Bull..

[8] Zhang H., Yuan Y., Li W., Li Y., Chai Y. (2016). Assessment of Three Tropospheric Delay Models (IGGtrop, EGNOS and UNB3m) Based on Precise Point Positioning in the Chinese Region. Sensors.

[9] Yao Y., He C., Zhang B., Xu C. (2013). A new global zenith tropospheric delay model GZTD. Chin. J. Geophys..

[10] Niell A.E. (1996). Global mapping functions for the atmosphere delay at radio wavelengths. J. Geophys. Res..

[11] Boehm J., Niell A., Tregoning P., Schuh H. (2006). Global mapping function (GMF): A new empirical mapping function based on data from numerical weather model data. Geophys. Res. Lett..

[12] Lagler K., Schindelegger M., Boehm J., Krasna H., Nilsson T. (2013). GPT2: Empirical slant delay model for radio space geodetic techniques. Geophys. Res. Lett..

[13] Boehm J., Moller G., Schindelegger M., Pain G., Weber R. (2015). Development of an improved empirical model for slant delays in the troposphere (GPT2w). GPS Solut..

[14] Niell A.E. (2001). Preliminary evaluation of atmospheric mapping functions based on numerical weather models. Phys. Chem. Earth..

[15] Boehm J., Werl B., Schuh H. (2006). Troposphere mapping functions for GPS and very long baseline interferometry from European Centre for Medium-Range Weather Forecasts operational analysis data. J. Geophys. Res..

[16] Yao Y., Yu C., Hu Y., Liu Q. (2015). Using Non-meteorological Parameters Tropospheric Delay Estimation Model for Accelerating Convergence of PPP. Geomat. Inf. Sci. Wuhan Univ..

[17] Xu A., Xu Z., Ge M., Xu X., Zhu H., Sui X. (2013). Estimating Zenith Tropospheric Delays from BeiDou Navigation Satellite System Observations. Sensors.

[18] Zhou C., Peng B., Li W., Zhong S., Ou J., Chen R., Zhao X. (2017). Establishment of a Site-Specific Tropospheric Model Based on Ground Meteorological Parameters over the China Region. Sensors.

[19] Andrei C., Chen R. (2009). Assessment of time-series of troposphere zenith delays derived from the Global Data Assimilation System numerical weather model. GPS Solut..

[20] Lu C., Zus F., Ge M., Heinkelmann R., Dick G., Wickert J., Schuh H. (2016). Tropospheric delay parameters from numerical weather models for multi-GNSS precise positioning. Atmos. Meas. Tech..

[21] Lu C., Li X., Zus F., Heinkelmann R., Dick G., Ge M., Wickert J., Schuh H. (2017). Improving BeiDou real-time precise point positioning with numerical weather models. J. Geod..

[22] Li X., Zhang X., Ge M. (2011). Regional reference network augmented precise point positioning for instantaneous ambiguity resolution. J. Geod..

[23] Zhang X., Zhu F., Li P., Zhai G. (2013). Zenith Tropospheric Delay Interpolation Model for Regional CORS Network Augmented PPP. Geomat. Inf. Sci. Wuhan Univ..

[24] Zhang H., Yuan Y., Li W., Zhang B., Ou J. (2017). A grid-based tropospheric product for China using a GNSS network. J. Geod..

[25] Chen Q., Song S. (2011). Assessment of ZTD derived from ECMWF/NCEP data with GPS ZTD over China. GPS Solut..

[26] Thayer G. (1974). An improved equation for the radio refractive index of air. Radio Sci..

[27] Wang J., Zhang L., Dai A., Van Hove T., Van Baelen J. (2007). A near-global, 8-year, 2-hourly data set of atmospheric precipitable water from ground-based GPS measurements. J. Geophys. Res..

[28] Ma Z., Chen Q., Gao D. (2012). Study on accuracy of ZTD and ZWD calculated from ERA-Interim data over China. J. Geod. Geodyn..

[29] Zhang Z. (2001). The Crustal Movement Observation Network of China. China Basic Sci..

[30] Shi C., Zhao Q., Geng J., Lou Y., Ge M., Liu J. Recent development of PANDA software in GNSS data processing. Proceedings of the International Conference on Earth Observation Data Procession and Analysis (ICEODPA).

[31] Gu S., Lou Y., Shi C., Liu J. (2015). BeiDou phase bias estimation and its application in precise point positioning with triple-frequency observable. J. Geod..

[32] Zhang W., Lou Y., Gu S., Shi C., Haase J., Liu J. (2016). Joint estimation of GPS/BDS real-time clocks and initial results. GPS Solut..

[33] Gong X., Gu S., Lou Y., Zheng F., Ge M., Liu J. (2018). An Efficient Solution of Real-Time Data Processing for multi-GNSS Network. J. Geod..

[34] Kouba J. (2008). Implementation and testing of the gridded Vienna Mapping Function 1 (VMF1). J. Geod..

[35] Lou Y., Zheng F., Gu S., Wang C., Guo H. (2016). Multi-GNSS precise point positioning with raw single-frequency and dual-frequency measurement models. GPS Solut..

